# Small bowel perforation by ingested fish bone in a post-gastric bypass patient: a case report

**DOI:** 10.1093/jscr/rjaf626

**Published:** 2026-05-24

**Authors:** Lina Berrada Dirhoussi, Pauline Curchod, Cinzia Tanda, Valentina Belfontali

**Affiliations:** Department of General Surgery, Hôpital Yverdon-les-Bains, Établissement Hospitalier du Nord Vaudois (eHnv), 1400, Switzerland; Department of General Surgery, Hôpital Yverdon-les-Bains, Établissement Hospitalier du Nord Vaudois (eHnv), 1400, Switzerland; Department of General Surgery, Hôpital Yverdon-les-Bains, Établissement Hospitalier du Nord Vaudois (eHnv), 1400, Switzerland; Department of General Surgery, Hôpital Yverdon-les-Bains, Établissement Hospitalier du Nord Vaudois (eHnv), 1400, Switzerland

**Keywords:** gastric bypass, foreign body ingestion, bowel perforation, fish bone, bariatric surgery complications

## Abstract

Gastric bypass effectively treats morbid obesity but may cause complications like anastomotic strictures and marginal ulceration. Bowel perforation from foreign body ingestion is rare. A 49-year-old woman with previous gastric bypass presented with acute abdominal pain. A fish bone perforated her small intestine, causing an acute abdomen. Emergency laparotomy was performed, successfully repairing all perforations. Patients with gastric bypass may have anatomical changes increasing their risk of foreign body-induced perforation. Clinicians should consider this possibility when assessing sudden abdominal pain. Awareness of uncommon causes of abdominal pain in post-gastric bypass patients is crucial for accurate diagnosis and treatment.

## Introduction

Severe obesity can be effectively treated with gastric bypass surgery, which frequently results in significant weight loss. However, it carries risks that require close monitoring. Anastomotic strictures and marginal ulceration are common complications, leading to abdominal pain, nausea, and vomiting. Other side effects include gastrogastric fistulas, internal hernias, dumping syndrome, and nutritional deficiencies, notably calcium, vitamin D, and B12, potentially resulting in anemia and osteoporosis. Rapid weight loss can also cause gallstones [1].

Bowel perforation is a particularly dangerous consequence, which can occur even without prior gastrointestinal issues. Foreign body ingestion is a rare cause of such perforation, highlighting the importance of considering this diagnosis in patients presenting with sudden abdominal pain post-bypass surgery. This report underscores the necessity for early detection and intervention to prevent adverse outcomes.

## Case report

A 49-year-old Black woman with a history of controlled hypertension, sickle cell anemia, and gastric bypass surgery performed eight years ago in Nice, France, presented to the emergency department of our institution with sudden epigastric and left flank pain lasting 12 hours. She described the pain as cramping with a burning sensation and an urge to defecate, unrelieved by Buscopan and paracetamol. She denied nausea, vomiting, fever, or changes in stool pattern. No recent endoscopy had been performed.

On examination, her abdomen was soft, with laparoscopic scars and tenderness in the left flank and renal angle. Rovsing’s, McBurney’s, Psoas, and Murphy’s signs were negative. Vital signs were stable, the Glasgow Coma Scale score was 15/15, and she was breathing comfortably and was afebrile. Despite administration of 150 micrograms of fentanyl, the pain persisted.

Laboratory investigations showed normal hemoglobin levels, with a mean corpuscular volume of 79 femtoliters and a mean corpuscular hemoglobin of 27 picograms, suggesting microcytic anemia associated with her sickle cell disease. There was also evidence of inflammation without leukocytosis, but with a slight increase in C-reactive protein to 15.5 milligrams per liter, while renal and hepatic function tests were within normal limits. Urinalysis revealed ketonuria, attributed to reduced oral intake, but was otherwise unremarkable.

**Figure 1:**
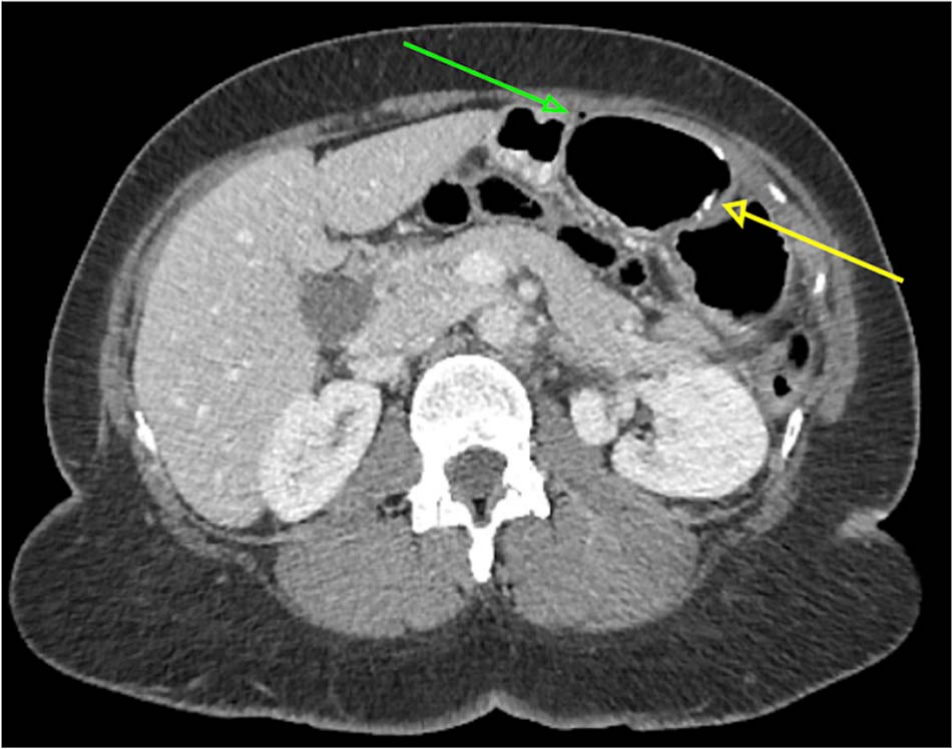
Axial abdominal CT scan showing a 4 cm hake fishbone (lower arrow) and free intraperitoneal air in the abdominal cavity (upper arrow).

**Figure 2:**
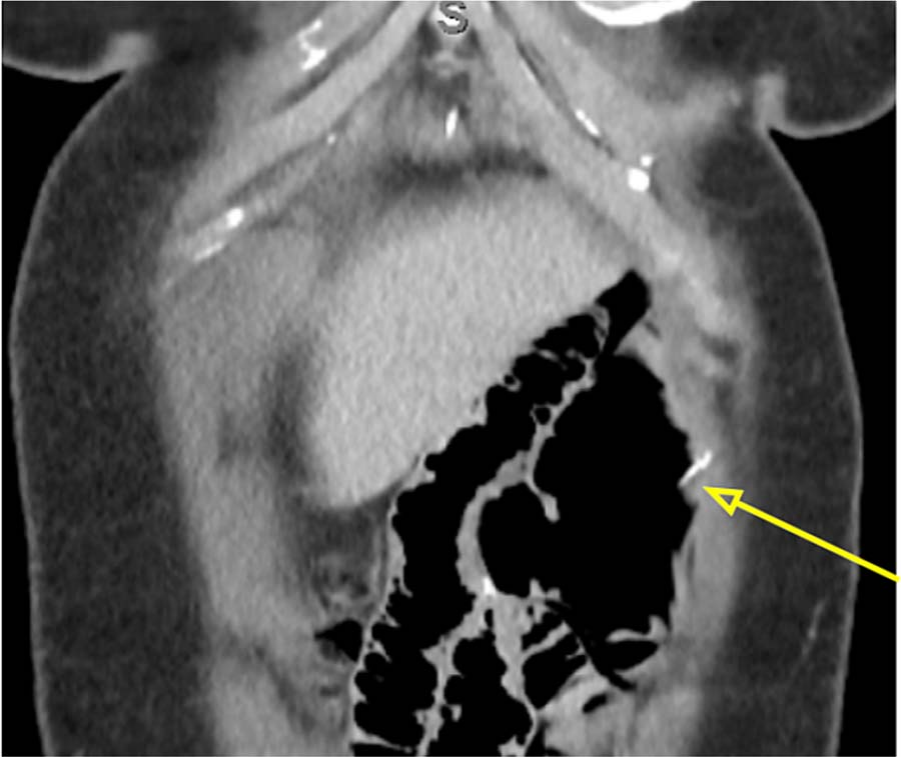
Coronal abdominal CT scan showing the 4 cm hake fishbone (arrow).

**Figure 3:**
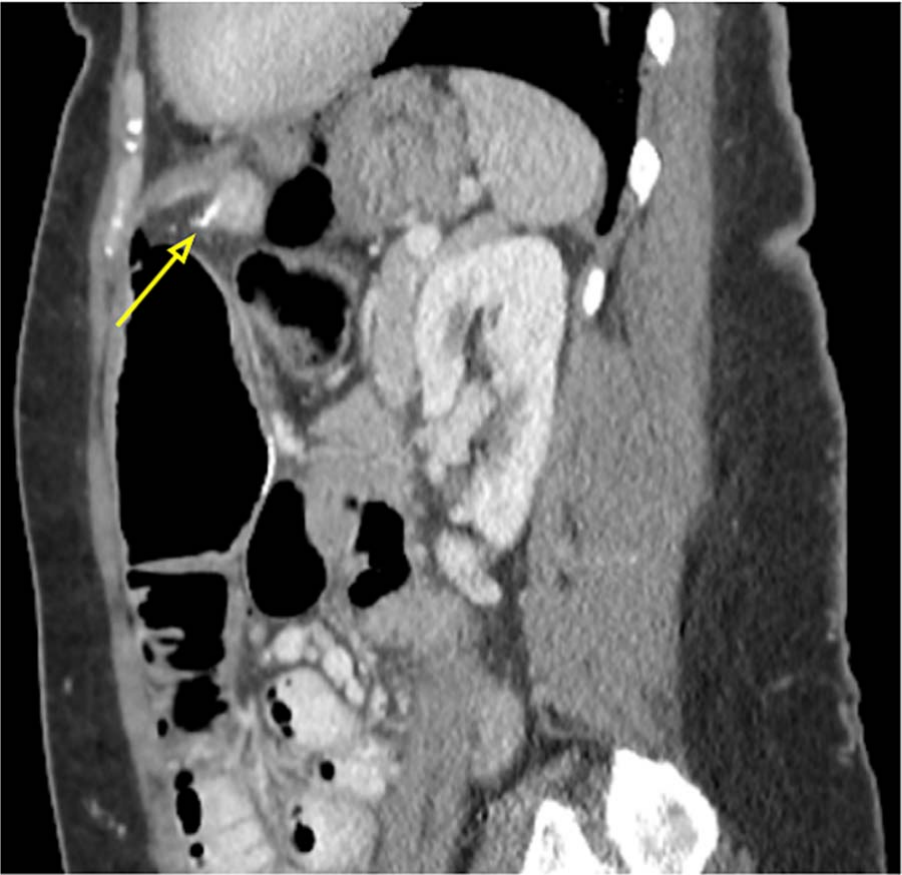
Sagittal abdominal CT scan showing the 4 cm hake fishbone (arrow).

Due to ongoing pain, a contrast-enhanced abdominal computed tomography (CT) was performed. It showed post-gastric bypass anatomy with no obstruction, but with an enlarged loop, free air in the left hypochondrium, and a 4 cm linear hyperdensity in the small bowel, consistent with a fish bone. No contrast extravasation was seen (Figs 1–3).

**Figure 4:**
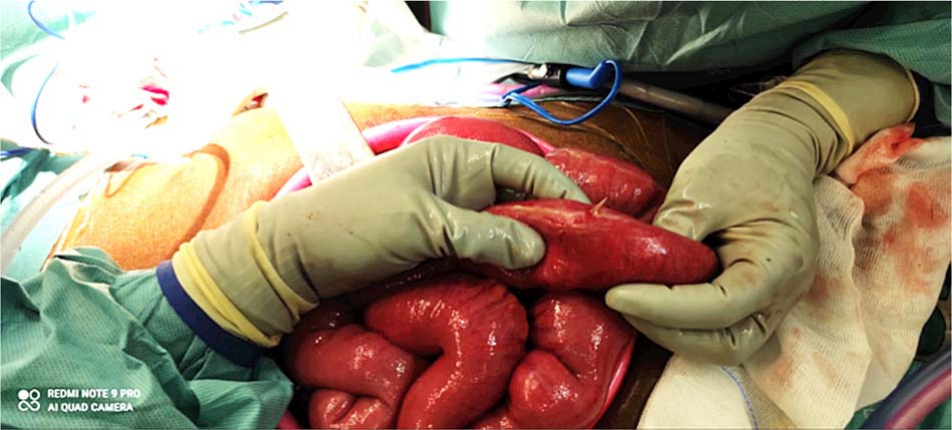
Intraoperative image of small bowel perforation caused by the hake fishbone.

**Figure 5:**
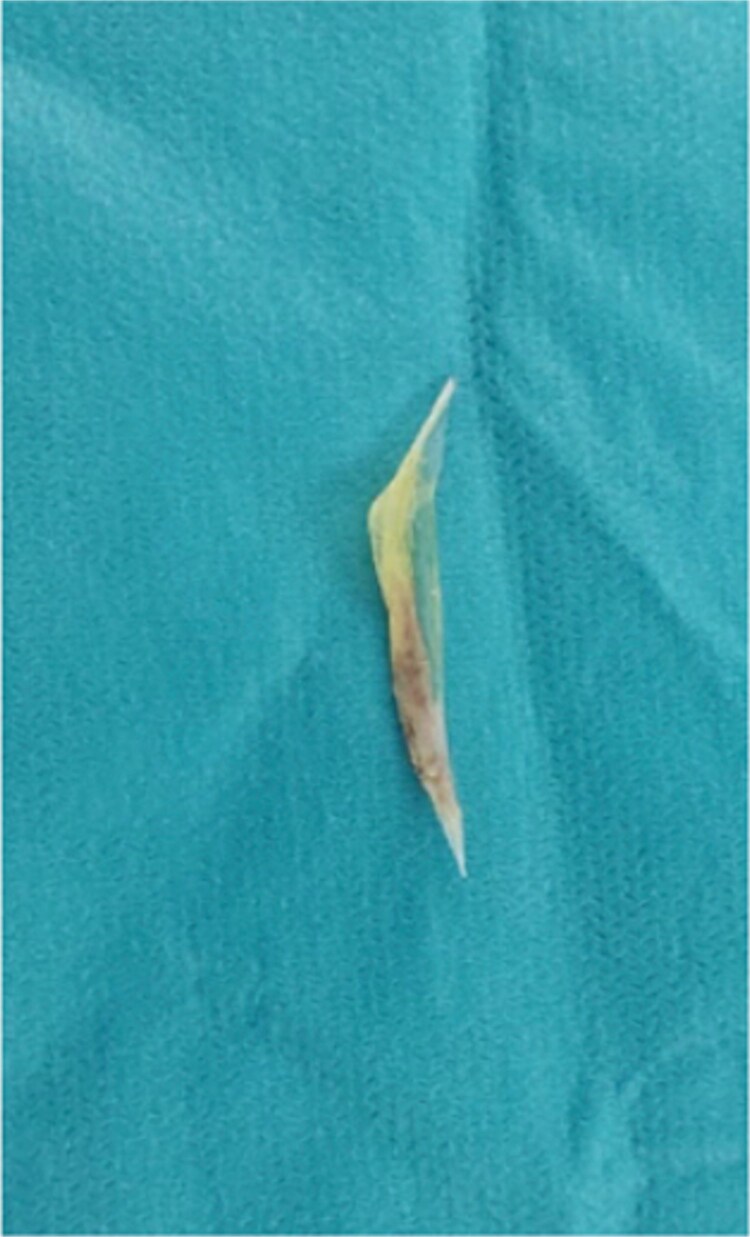
Extracted 4 cm hake fishbone responsible for the double small bowel perforation.

Surgical exploration was indicated. Diagnostic laparoscopy revealed mild small bowel dilatation and localized peritonitis, with turbid fluid in the Douglas pouch but no purulent collection. A hyperemic and fibrin-covered segment was noted near the biliary anastomosis. As the perforation could not be identified laparoscopically, conversion to a minilaparotomy was necessary. Two small bowel perforations caused by a 4 cm fish bone were identified near the foot of the alimentary limb. The foreign body was removed through a 0.5 cm enterotomy. Both perforations were closed using 3-0 Vicryl sutures, followed by peritoneal lavage and omental patching (Figs 4 and 5). The patient recovered uneventfully and later recalled eating hake, although she did not remember ingesting a bone.

## Discussion

Foreign body ingestion is common, but <1% of cases result in bowel perforation—most commonly caused by sharp objects like fish bones, chicken bones, or toothpicks. Fish bones account for up to 84% of such cases [2]. In adults, accidental ingestion is much more common than intentional ingestion, which is typically associated with prisoners or suicidal patients [3].

Unintentional ingestion is also more frequent among elderly individuals, children, and those with psychiatric disorders or impaired vision. Predisposing factors include cold beverage consumption, NSAID use, rapid eating, and poor dentition [4]. Although ~75% of ingested objects become lodged in the cricopharyngeal sphincter, >90% of objects that reach the stomach pass without complications. Endoscopic retrieval is required in 10%–20% of cases, whereas surgery is necessary in approximately 1% [5].

Anatomically, the duodenal C-loop and terminal ileum are the most vulnerable due to fixed angulations and limited mobility [6]. Post-bypass patients may be more susceptible due to altered intestinal architecture, including angulated loops and slower transit. These features can increase the likelihood of obstruction or perforation [1].

The intestinal mucosa normally defends itself through mucus production and peristalsis. However, sharp foreign bodies such as fish bones can anchor into weak points, causing necrosis or inflammation [7, 8]. Most pass within a week [1, 4], but lodged bones can result in delayed injuries.

Diagnosis is difficult because symptoms can mimic appendicitis, diverticulitis, or perforated ulcers [9]. Patients may present with sharp pain, rebound tenderness, fever, nausea, vomiting, or hemodynamic instability [10].

Imaging is essential in the evaluation of suspected foreign body ingestion and its complications. Plain radiographs can detect metallic objects and free intraperitoneal air but often fail to identify nonmetallic materials such as fish bones [11]. Ultrasound may reveal localized fluid collections but is less reliable for evaluating deep abdominal structures. CT is the imaging modality of choice, as it can demonstrate fish bones, pneumoperitoneum, bowel wall thickening, and fat stranding [12]. Multidetector CT (MDCT) provides high-resolution, multiplanar reconstructions that enhance detection of nonmetallic foreign bodies [13]. In addition to confirming the presence of a foreign body, CT helps identify complications and guide the need for surgical intervention. Radiographic opacity of fish bones can vary by species, which may limit detection on plain films, whereas CT can reveal associated tissue reactions and penetration depth. Ultra-low-dose CT offers these diagnostic advantages while reducing radiation exposure [12]. In the present case, CT established the diagnosis and enabled prompt surgical management.

While contrast esophagography is not frequently used due to the risk of aspiration, it can still be valuable in detecting esophageal perforations. This procedure should use water-soluble contrast agents, which are quickly absorbed, minimizing complications [12, 14].

Prompt removal of fish bone foreign bodies is essential due to the risk of perforation (15%–35%) [15]. They are often retrieved successfully within 6 hours if lodged above the cricoid. Flexible endoscopy is generally preferred for retrieval because it avoids general anesthesia and has lower complication rates. Rigid endoscopy is more effective for objects lodged in the upper esophagus or with complex anatomy.

Once perforation occurs, surgical intervention is typically required. Laparoscopy is less invasive and preferred when feasible, but conversion to open surgery may be necessary if the perforation is not located. Management options include enterotomy, primary suture, or resection, depending on perforation size, contamination, and bowel viability [16]. Our patient underwent minilaparotomy for foreign body removal and successful suture closure, with a good clinical outcome.

This case highlights the importance of considering rare but serious causes of abdominal pain in post-gastric bypass patients. Altered anatomy increases susceptibility to complications from ingested objects. CT is indispensable for diagnosis, especially from radiolucent materials like fish bones. Early detection and individualized surgical management remain essential to reduce morbidity and ensure favorable outcomes.

## Data Availability

The data generated or analyzed during this study are fully included in this article. For further inquiries, please contact the corresponding author.
